# Initial systolic blood pressure and ongoing internal bleeding following torso trauma

**DOI:** 10.4103/0974-2700.76833

**Published:** 2011

**Authors:** Daniel S Kassavin, Yen-Hong Kuo, Nasim Ahmed

**Affiliations:** Department of Surgery, Monmouth Medical Center Long Branch, NJ, USA; 1 Department of Biostatistics, Jersey Shore University Medical Center Neptune NJ, USA; 2 Department of Surgery, Division of Trauma and Surgery Critical Care, Jersey Shore University Medical Center, Neptune NJ, USA

**Keywords:** Initial systolic blood pressure, internal bleeding, hemodynamic stability

## Abstract

**Objective:**

Recent studies have suggested that an initial systolic blood pressure (SBP) in the range of 90–110 mmHg in a trauma patient may be indicative of hypoperfusion and is associated with poor patient outcome. However, the use of initial SBP as a surrogate for predicting internal bleeding is yet to be validated. The purpose of this study was to assess the presenting SBPs in patients with torso trauma and evidence of ongoing internal hemorrhage.

**Setting and Design:**

This was a retrospective chart review conducted at the Level II Trauma Center.

**Patients and Methods:**

Adult patients who sustained trauma and underwent chest and/or abdominal computed tomography (CT) scans and angiography were included in the study. Demographic and clinical information was extracted from patients who had CT scan and angiography. Extravasation of contrast material on CT scan and angiography was considered positive for ongoing internal bleeding.

**Results:**

From January 2002 through July 2007, a total of 113 consecutive patients were included in this study. Forty-seven patients had evidence of ongoing internal bleeding (41.6%; 95% confidence interval: 32.4%, 51.2%). When comparing patients with and without ongoing bleeding, these two groups were similar in their gender, race, pulse, injury severity score and shock index. However, bleeding patients were typically older [mean (standard deviation): 44.5 (20.5) vs 37.3 (19.1) years; *P* = 0.051], had a lower initial SBP [116.2 (36.0) vs 130.0 (30.4) mmHg; *P* = 0.006] and had a higher Glasgow coma scale (GCS) [13.1 (4.0) vs 12.1 (4.4); *P* = 0.09]. From a multivariate logistic regression analysis, older age (*P* = 0.046) and lower SBP (*P* = 0.01) were significantly associated with bleeding, when controlled for gender, race and GCS. Among the 47 patients with ongoing bleeding, only seven patients (15%) had a SBP lower than 90 mmHg and 25 patients (53%) had a SBP higher than or equal to 120 mmHg. The spleen was the most frequently injured organ identified with active bleeding.

**Conclusions:**

Initial SBP cannot predict the ongoing internal bleeding.

## INTRODUCTION

Systolic blood pressure (SBP) is a clinical marker used as an indicator of trauma severity. It plays a key role in clinical management and in determining outcomes and has been incorporated into status scores such as the Revised Trauma Score (RTS) and the Trauma and Injury Severity Score (TRISS).[1]

SBP of 120 mmHg is considered normal in adults.[2] In a trauma patient, hypotension has traditionally been defined as a SBP below 90 mmHg.[1] The American College of Surgery Committee on Trauma recommends that injured patients should be transported to a designated trauma center if the SBP is <90 mmHg.[3]

The body’s physiologic response to trauma is to release endogenous catecholamines, which results in an increase in heart rate and systemic vascular resistance despite the ongoing blood loss.[4] The cumulative effects result in an increase in initial blood pressure. Therefore, one would expect trauma patients who sustain minimal loss of blood volume and remain free of ongoing blood loss to have blood pressure values above that of the general population at large. Thus, the use of a cutoff of 90 mmHg for the lower limits of normal SBP is too low.

A study reviewing the SBP from the National Trauma Data Bank (NTDB) determined that with a SBP of 110 mmHg and lower, there was a significant increase in mortality such that for every 10 mmHg decrease in SBP, there was an associated 4.8% increase in mortality. This was relative to a baseline mortality rate of less than 2.5%[6]

Our study assesses the initial SBP in patients who presented with ongoing internal bleeding diagnosed on computed tomography (CT) and/or angiogram. We hypothesized that the current normal reference ranges of SBP in the trauma patient are perhaps inaccurately low, resulting in the false stratification of unstable patients who may be hemorrhaging into the normal group. This may result in disastrous consequences as trauma patients who present with incrementally decreasing physiological reserve due to ongoing internal bleeding are inappropriately triaged.

## PATIENTS AND METHODS

Jersey Shore University Medical Center (JSUMC) is a Level II Trauma Center located in central New Jersey. This study was a retrospective review of the JSUMC Trauma Registry from January 2002 till July 2007. The study was approved by the Institutional Review Board.

### Patients’ characteristics

All adult patients who sustained chest and/or abdominal traumas and underwent multidetector computed tomography (MDCT) scan and follow-up angiogram as part of their diagnostic work up were included in this study. The decision to perform CT scan was based on the attending trauma surgeon’s clinical evaluation for suspected injuries to the chest and/or the abdominal cavities. Angiography was then performed in those patients whose MDCT scan demonstrated either arterial blush (high attenuation compared to surrounding structures) or high grade injuries without evidence of blush for both diagnostic and therapeutic purposes. Hemodynamically unstable patients who did not respond to initial fluid boluses or blood transfusions and required immediate surgery were excluded from the study. 113 consecutive trauma patients who met the above criteria were included in this study.

Patients’ demographics and clinical information were extracted from the Trauma Registry and medical records. CT scans and angiography findings were reviewed. Patients who had negative results on both CT scan and angiography were defined as not bleeding.

### Initial systolic blood pressure measurement

Initial SBP was measured in all patients in trauma bay using noninvasive blood pressure monitoring device, infinity modular monitoring series (Draeger Medical System, Inc., Denver MA, USA). A pneumatic cuff was wrapped around the arm or leg and a hose linked the cuff to the monitor. Blood pressure was determined by inflation and deflation of the cuff using oscillometric method.

### Computed tomography scan

CT scans of the chest, abdomen and pelvis were performed from the thoracic inlet to the pubic symphysis, whereas abdominal and pelvic scans were done from the diaphragm to the pubic symphysis. During the last 2 years of this study period, a Phillips 64-slice MDCT scan was used. Prior to this, a GE 4-slice MDCT was utilized. Portal venous phase CT scan was obtained with 5 mm slices by injecting 100 ml of contrast, Omnipaque 300 or Visipaque if creatinine was elevated, at a rate of 2–4 ml/second, depending upon the peripheral access. Kidney delays, when ordered, were obtained after 3 minutes. High attenuation density as compared to surrounding tissue and vascular structures was considered positive for arterial bleeding.[7] No oral contrast was administered.

### Angiogram

Angiogram was performed in a Philips digital subtraction angiography suite via a common femoral artery approach. For the aortogram, 20 ml contrast was used at a rate of 10 ml/second and for solid organ vessels (such as the liver and spleen) 10 ml at a rate of 5 ml/second was used. Extravasation of contrast was considered positive for ongoing bleeding. Patients who were tested positive for ongoing internal bleeding underwent angioembolization using coil, gelfoam or polyvinyl alcohol.

### Statistical analysis

Mean (standard deviation) and percentage were used to summarize patient characteristics. The Fisher exact test or chi-square test was used to compare proportions. The Wilcoxon rank sum test was used to compare the continuous variables because the normality assumption was not met. Multivariate logistic regression analysis was performed to assess the association between bleeding and clinical factors.

## RESULTS

In our review, 113 consecutive trauma patients underwent both CT and angiography in the course of their presenting workup. The average time at which the bleeding and nonbleeding patients arrived to the trauma center directly from the scene was 49.3 and 46.8 minutes, respectively. This was the exact time when the medical dispatcher received the 911 call from the field and the patients arrived at the Trauma Admitting Area. Forty-seven patients (41.6%; 95% confidence interval: 32.4%, 51.2%) were identified with ongoing bleeding. Forty-one of 47 patients were successfully managed with only angioembolization, whereas the other six patients required additional operative intervention. The characteristics of patients with and without diagnosed extravasation are shown in Table 1. The most frequently identified organ systems with evidence of extravasation on CT were the spleen, pelvis and liver. The majority of these injuries were ≥3 on the Abbreviated Injury Scale.

**Table 1:** Patient characteristics

Characteristic	No bleeding (*n* = 66)	Bleeding (*n* = 47)	*P* value
Female	30%	38%	0.49
Age	37.3 (19.1)	44.5 (20.5)	0.051
Race			0.76
African American	12%	9%	
Caucasian	88%	91%	
Time (minutes) dispatch PH to TAA*	46.8 (15.5)	49.3 (17.0)	0.61
Pulse	100.1 (23.8)	93.5 (21.6)	0.21
SBP	130.0 (30.4)	116.2 (36.0)	0.006
SBP at scene*	125.1 (32.3)	112.3 (32.2)	0.02
ISS	26.2 (13.0)	25.4 (14.9)	0.48
GCS total	12.1 (4.4)	13.1 (4.0)	0.09
Shock index	0.79 (0.26)	0.85 (0.39)	0.52

* PRE-HOSPITAL DATA OF ONLY 56 PATIENTS IN NO BLEEDING AND 36 PATIENTS IN THE BLEEDING GROUPS WERE AVAILABLE; DISPATCH PH: RECEIVING OF 911 CALL FROM THE SCENE OF INJURY TO PREHOSPITAL PERSONAL; TAA: TRAUMA ADMITTING AREA

Patients with active extravasation (bleeding) had a statistically significant lower SBP. They also tended to be older and have a higher Glasgow coma scale (GCS). There is no statistically significant difference between both the groups with regard to gender, race, heart rate, injury severity score and shock index.

**Figure 1:**
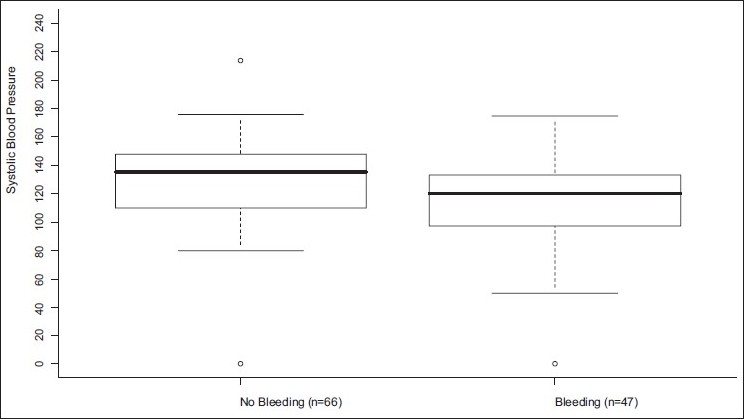
Distributions of the SBP by bleeding status

**Figure 2:**
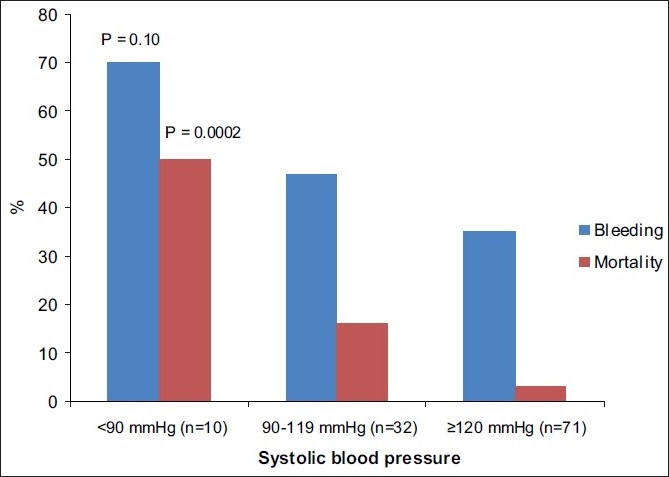
Relationship of initial SBP to internal bleeding and mortality

When the SBP of 120 was considered as a cut point, 53% of the bleeding patients were found to have SBP ≥ 120 [Figure 1]. When patients with active extravasation on CT were stratified into initial blood pressure categories (<90 mmHg, 90–19 mmHg, ≥120 mmHg), only seven patients (15%) had an SBP lower than 90 mmHg while 25 patients (53%) had an SBP greater than or equal to 120 mmHg. The chance of death was much higher in the lowest SBP group (*P* = 0.0002). The chance of bleeding tended to be higher in the lowest SBP group (*P* = 0.10)[Figure 2].

On performing a multivariate logistic regression analysis, active extravasation was associated with an older age (*P* = 0.046) and lower SBP (*P* = 0.01) when controlled for race and GCS.

## DISCUSSION

Hypotension in the trauma patient is a late clinical finding and is defined as Class III hypovolemic shock, representing 30–40% loss of total blood volume. It is associated with high mortality.[8,9] However, patients with less severe blood loss may initially present with a “normal” blood pressure but subsequently progress to irreversible shock if appropriate intervention is not performed in a timely fashion. To consider an initial SBP of 120 mmHg in a trauma patient with multiple injuries as “normal” may have dire consequences if the patient continues to have ongoing internal bleeding. This problem has the potential to be compounded by the ubiquitous use of automated blood pressure cuffs which have been found to overestimate blood pressures in those patients with blood pressures ranging from 91 to 110 mmHg.[10] SBP is the pressure exerted in the arteries at the end of the cardiac cycle when ventricles are contracting. It varies with each heart beat and is affected by the blood volume as well. Using the noninvasive monitor appropriate cuff size is an important factor for the measurement of accurate blood pressure. However, most commonly used device for blood pressure measurement in emergency setting and also in our practice was noninvasive blood pressure system device using the oscillometric method. This system may produce some error in blood pressure reading in some patients due to heart and circulation problem.

Wong *et al*. observed that a SBP of <100 mmHg was the most important variable in determining failed observation during nonoperative approaches in patients with solid abdominal organ injuries.[11] Their conclusion was that a SBP of <100 mmHg in patients with active extravasation should be defined as a failure of conservative management and that these patients necessitate either surgical or radiological intervention. In our study, 42 patients presented with a blood pressure below 120 mmHg, of whom 21 demonstrated extravasation on CT, necessitating 19 embolizations.

Trauma victims with slow ongoing internal bleeding can produce either biphasic or triphasic responses, depending upon the total amount of blood loss.[4] Typically, small quantities of blood loss (10–15% of total blood loss) can lead to a decrease in firing of arterial baroreceptor, which results in an increase in sympathetic response to heart, and in peripheral vascular resistance and a decrease in cardiac vagal response. This cumulative effect leads to an initially increased heart rate and SBP. If the blood loss continues, it will lead to activation of “depressor” reflex which overrides the baroreceptor reflex and causes bradycardia and hypotension (biphasic response). Once bleeding continues to around 40% of total blood volume, it will lead to a massive increase of heart rate with profound hypotension (triphasic response) which is likely the irreversible phase of hemorrhagic shock. Fluid resuscitation may blunt this typical response, especially at the initial state of injury and bleeding. In our study, there was significant difference in the SBP at the scene and in the Trauma Admitting Area. Bleeding patients tend to have lower SBP. However, there was a slight increase in SBP of bleeding and nonbleeding patients in the trauma room. Perhaps, the difference in SBP was due to early initiation of fluid resuscitation at the scene.

It is well documented that hypotension (SBP ≤ 90 mmHg) in trauma patients either on the field or in the emergency department is associated with a higher injury severity score (ISS) score, higher in-hospital mortality, increased requirements for emergency laparatomies, blood transfusions, intensive care unit (ICU) days, ventilator days and total hospital stays.[12–15] However, there have been only a few recent studies that have put to question the definition of normotension in the trauma patient. Bruns and colleagues analyzed several pre-hospital SBP levels and their impact on outcome.[16] In their study, pre-hospital SBP was strongly correlated with the emergency room SBP. The in-hospital mortality was 10% in patients with a pre-hospital SBP of 111–120 mmHg and the risk of mortality sharply increased below 110 mmHg SBP. They concluded that using a cutoff for pre-hospital SBP of 110 mmHg was a more appropriate threshold for defining hypotension and was more appropriate for determining trauma center triage. Eastridge presented the data of more than 700,000 patients from the NTDB and correlated emergency department SBP and base deficit (BD) as a marker of hypoperfusion with in-hospital mortality as an outcome.[6] Hypoperfusion (defined as BD > 4.5 from the baseline) begins to increase with an SBP of ≤118 mmHg. Similarly, the mortality increased by 4.8% from the baseline with every 10 mmHg decrease in SBP at 110 mmHg. In our series, patients with an intermediate blood pressure range (90–119 mmHg) were found to have a mortality rate almost six times higher than those patients with a blood pressure of 120 mmHg or higher. Similar findings were noted in a study by Edelman and Moore as well.[17]

Based on the current definition of normotension, our results showed that even if the initial SBP ≥ 120 was used for the cut point, 35% of the patients in that group showed internal bleeding, which represents a substantial number of patients who would have patenti been triaged to a rather stable group.

Furthermore, the data of all the patients who were identified with internal bleeding on CT scan and/or angiogram either in the chest, abdominal and/or pelvic cavities and correlated with the initial SBP, 53% of bleeding patients presented with an initial SBP at ≥120 mmHg. This and other studies raise many questions as to what should be considered the normal lower limits of SBP in trauma patients. Is the current reference range which stratifies the majority of patients with active internal hemorrhage into a stable group accurate? It appears that the current definition of normotension puts patients at increased risk of morbidity and mortality.

Limitations of this study include the fact that it is a retrospective study and is reflective of a single institution and the experiences of its affiliated group of trauma surgeons.

## CONCLUSIONS

Although a patient who is defined as “normotensive” following torso trauma can have ongoing internal bleeding, an initial SBP of less than 90 mmHg in this patient population poses a significant risk of ongoing bleeding and poorer outcome.

Therefore, it is imperative to re-evaluate the current cut-off value used to define the term “normotensive”.
